# An Integrated Nutritional and Physical Activity Approach for Osteosarcopenia

**DOI:** 10.3390/nu17172842

**Published:** 2025-08-31

**Authors:** Edoardo Mocini, Ludovica Cardinali, Olivia Di Vincenzo, Antimo Moretti, Carlo Baldari, Giovanni Iolascon, Silvia Migliaccio

**Affiliations:** 1 Department of Theoretical and Applied Sciences, eCampus University, 22060 Novedrate, Italy; carlo.baldari@uniecampus.it; 2 Department of Life Science, Health, and Health Professions, Link Campus University, 00165 Rome, Italy; l.cardinali@unilink.it; 3 Department of Experimental Medicine, University Sapienza of Rome, 00185 Rome, Italy; olivia.divincenzo@uniroma1.it (O.D.V.); silvia.migliaccio@uniroma1.it (S.M.); 4 Department of Medical and Surgical Specialties and Dentistry, University of Campania “Luigi Vanvitelli”, 80138 Naples, Italy; antimo.moretti@unicampania.it (A.M.); giovanni.iolascon@unicampania.it (G.I.)

**Keywords:** osteosarcopenia, osteosarcopenic obesity, bone–muscle axis, chronic inflammation, nutritional intervention, physical function, multidisciplinary approach

## Abstract

Osteoporosis is a skeletal disorder characterized by decreased bone strength, which leads to an increased risk of developing fractures. Interestingly, this metabolic disorder is often related to sarcopenia, defined as decreased muscle mass, strength, and function. These two conditions appear to be closely connected, leading to a clinical condition named osteosarcopenia (OS). Aging may explain the link between muscle and bone loss through genetic, mechanical, endocrine, and nutritional factors. Further, aging increases the amount of adipose tissue, often due to sedentary behavior and unbalanced nutritional pattern, leading to a clinical condition defined as osteosarcopenic obesity, characterized by concurrent obesity, sarcopenia, and osteoporosis, where each condition exacerbates the others. Moreover, sarcopenia leads to decreased physical (PA) activity, worsening skeletal homeostasis, and creating a vicious cycle, which increases falls, fracture risk, and disability. This review underscores the importance of a systemic approach, focusing on nutritional therapy integrated with PA and, eventually, pharmacological interventions to efficiently manage (OS).

## 1. Introduction

Osteoporosis is a skeletal disorder characterized by decreased bone strength, which leads to an increased risk of developing fractures [1]. Osteoporosis is often linked to sarcopenia, which can be defined as a decrease in muscle mass, function, and strength. These two chronic conditions are closely interconnected and can be conceptualized as a single syndrome, osteosarcopenia (OS), which contributes to musculoskeletal fragility, leading to a greater number of falls, fractures, reduced quality of life, and decreased survival [2]. Aging might explain the link between muscle and bone loss through genetic, mechanical, endocrine, and nutritional factors. Moreover, aging is linked to an increased amount of adipose tissue, due to sedentary behavior and unbalanced nutritional pattern, often leading to a clinical condition defined as osteosarcopenic obesity (OSO) [3]. This syndrome is characterized by concurrent obesity, sarcopenia, and osteoporosis, with each condition contributing to the onset and worsening of other clinical conditions, due to a loss of homeostatic balance linked to an altered cytokine pattern, as well as poor nutrition and scarce exercise. In particular, the increase in adipose tissue might induce chronic low-grade inflammation and adipokine dysregulation, which accelerate both muscle degradation and bone resorption [4]. At the same time, sarcopenia leads to decreased physical activity (PA), impairs skeletal homeostasis, increases fracture risk and disability, and contributes to the development of a frailty syndrome [5]. Indeed, research has identified the “bone–muscle axis” as a critical framework for understanding OS, emphasizing the interconnection of these tissues via molecular mediators such as myokines and osteokines [2,3,4,5,6]. Thus, this review underscores the importance of a systemic approach, focusing on nutritional therapy integrated with PA and, eventually, pharmacological interventions to efficiently manage OS.

## 2. Methods

### 2.1. Narrative Review Construction

The present narrative review was conducted following the principles of the “Narrative Review Checklist” proposed by the Academy of Nutrition and Dietetics [7]. This approach guided the organization of the manuscript, ensuring a coherent structure and the careful selection of studies relevant to the topic.

### 2.2. Studies Selection

The literature search was conducted in three electronic databases: PubMed, Scopus, and Google Scholar. To complete the research and ensure saturation of the literature, references of selected articles were also considered. The search strategy included all types of English-language articles without publication period restriction. The search terms were “osteoporosis”, “sarcopenia”, “osteosarcopenia”, “sarco-osteopenia” combined with “bone mass loss”, “muscle mass loss”, “bone mass density”, “muscle strength”, and “exercise”, “physical activity”, “physical fitness”, “resistance training”, “aerobic exercise” and “nutrition”, “protein supplements”, “Vitamin D”, “Calcium”.

This review included both observational cohort, case-control, cross-sectional studies, systematic reviews, and meta-analyses, and randomized, double-blind studies (randomized controlled trials, RCTs). Finally, editorials, letters to the editor, and conference proceedings were excluded from the review.

## 3. Conceptualizing OS as a Unified Syndrome

**Figure 1:**
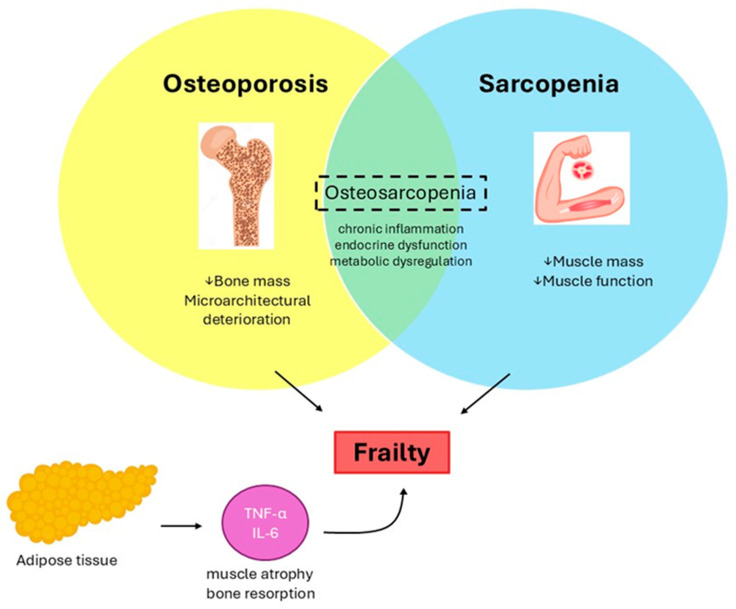
Conceptual framework of osteosarcopenia as a unified syndrome. Adapted from Salamanna, F. et al. [17].

OS should be conceptualized as a unique syndrome, considering that osteoporosis and sarcopenia share common pathophysiological mechanisms (Figure 1), which include chronic inflammation, endocrine dysfunction, and metabolic dysregulation [8]. The concept of OS as a unified syndrome highlights its systemic nature, emphasizing the need to treat sarcopenia and osteoporosis as a single, interconnected condition. This concept is further supported by the fact that the increase in adipose tissue, often observed during the aging process, leads to a subclinical inflammatory state, characterized by increased levels of pro-inflammatory cytokines, such as tumor necrosis factor-alpha (TNF-α) and interleukin-6 (IL-6), which exacerbate muscle atrophy and bone resorption [9,10]. Also, body composition modification occurring with aging can significantly affect the interplay among bone, muscle, and fat tissues, considering that adipokines, such as leptin and adiponectin, influence bone metabolism and muscle function, while myokines and osteokines contribute reciprocally to adipose tissue regulation.

For instance, irisin, a myokine secreted during exercise, has been shown to promote osteoblast activity and reduce adipogenesis, highlighting the interconnected regulatory networks among these mesenchymal-derived tissues [11,12]. From a clinical perspective, sarcopenia is associated with, and contributes to, decreased PA, crucial for maintaining optimal bone strength and metabolic health. Additionally, osteoporosis contributes to frailty, exacerbating sarcopenia in patients with fragility fractures, due to decreased mobility [13]. This complex interplay underscores the need for an integrative approach in both research and clinical practice. Social determinants of health, including socioeconomic status, access to high-quality nutrition, availability of safe environments for PA, and educational disparities can significantly contribute to the development and maintenance of OS. From a biopsychosocial perspective, individuals experiencing socioeconomic disadvantage not only face physical barriers to preserving a balanced diet and engaging in regular exercise but may also encounter psychological stressors and limited health literacy [14]. These factors can negatively influence health behaviors and adherence to preventive or therapeutic interventions, further increasing vulnerability to both muscle loss and reduced bone density. Addressing OS requires a comprehensive, multidimensional approach that considers not only biological risk factors but also the social and psychological context influencing individuals’ daily lives and aging. This approach should promote multidisciplinary therapeutic strategies that simultaneously target muscle and bone tissues [14]. Critics may argue that conceptualizing OS as a unified syndrome risks oversimplifying the condition or overlooking condition-specific characteristics. However, this integrative perspective preserves the need for tailored interventions while emphasizing the systemic nature of OS. It supports treatment strategies that target shared pathophysiological pathways, yet remain adaptable to individual variability [15,16]. Moreover, comprehensive approaches can help to avoid the pitfalls of managing conditions separately, such as implementing therapeutic strategies for obesity that fail to preserve lean mass and bone density, thereby potentially worsening both sarcopenia and osteoporosis.

## 4. The Role of Mitochondria in Muscle–Bone–Metabolic Health

Mitochondria are central regulators of cellular energy production and redox balance, playing a pivotal role in both muscle and bone homeostasis. In skeletal muscle, mitochondria provide ATP through oxidative phosphorylation to sustain contractile activity, protein synthesis, and repair processes. In bone tissue, osteoblasts rely on mitochondrial function to support the energetically demanding process of bone matrix production and mineralization.

Aging-related mitochondrial dysfunction, characterized by reduced number, decreased enzyme activity, mutations in mitochondrial DNA, and increased morphological abnormalities, is closely associated with sarcopenia, through impaired energy production and elevated oxidative stress [18].

In particular, mitochondria are essential in osteoblasts for matrix production and mineralization. Mitochondrial dysfunction alters the equilibrium between osteogenesis and osteoclast activity, thus contributing to osteoporosis [19].

In bone, mitochondrial deficiency in osteoblasts reduces their differentiation and mineralizing capacity, while increased ROS promotes osteoclast activity, thereby shifting bone remodeling toward resorption [19].

Mitochondrial health is also linked to metabolic health. Efficient mitochondria enhance lipid and glucose oxidation, improving insulin sensitivity and reducing systemic inflammation, both critical for preserving muscle mass and bone density [20]. Conversely, mitochondrial dysfunction contributes to metabolic inflexibility, ectopic fat accumulation, and chronic low-grade inflammation, which further impair musculoskeletal integrity [20].

Physical activity exerts a profound influence on mitochondrial health, with key implications for both muscle and bone metabolism. In skeletal muscle, physical exercise, particularly resistance and endurance, stimulates mitochondrial biogenesis, improves mitochondrial dynamics, enhances oxidative capacity, and strengthens redox balance. These adaptations collectively compromise the trajectory of age-related sarcopenia and support metabolic homeostasis. Pahlavani et al. reported that exercise increases mitochondrial biogenesis by activating genes affected by PGC1-ɑ (such as CaMK, AMPK, MAPKs) and altering cellular calcium, ATP-AMP ratio, and cellular stress by reconstituting a healthier mitochondrial network [21]. The authors state that moderate-intensity exercise can be used as a non-invasive treatment for sarcopenia by activating pathways that regulate the mitochondrial network in skeletal muscle. A systematic review by Long et al. (2022) confirms that physical exercise during aging regulates key mitochondrial quality control mechanisms, including biogenesis, fusion, fission, and mitophagy, thereby attenuating sarcopenia [22]. Consistently, Wang et al. recently reported that exercise training preserves mitochondrial homeostasis by enhancing mitophagy, maintaining a balanced dynamic between fusion and fission processes, and sustaining redox equilibrium [23]. Interestingly, exerkines, exercise-induced signaling molecules, seem to play a pivotal role in mediating these mitochondrial benefits [23]. Moreover, a recent systematic review and meta-analysis by Abrego-Guandique et al. demonstrated that endurance exercise consistently upregulates the expression of PGC-1α, a master regulator of mitochondrial biogenesis, with large positive effect sizes across randomized trials, underscoring the robust stimulus that physical activity exerts on mitochondrial pathways [24]. Nutritional strategies can also promote mitochondrial biogenesis and protect against oxidative damage.

Thus, exercise and nutrition interventions targeting mitochondrial preservation represent a promising avenue for integrated prevention and treatment of OS.

## 5. Nutritional Approach

### 5.1. Protein Intake

Adequate protein intake plays a central role in the management of both osteoporosis and sarcopenia, and also potentially obesity-associated complications. Indeed, protein is crucial not only for muscle preservation but also for mitigating inflammation and supporting bone remodeling, both of which are critical in the context of OS [13,25]. While in an adult optimal protein intake is usually defined within 0.9 to 1.0 g/kg of ideal body weight/day, the recommended protein intake might raise to 1.2–1.6 g/kg of body weight per day, as indicated by the ESPEN working group, with higher levels potentially needed in older adults to overcome anabolic resistance, the body’s decreased ability to efficiently use dietary proteins to promote muscle protein synthesis (MPS) [26,27]. Furthermore, evidence suggests that higher protein levels, up to 2.0 g/kg/day, might be necessary during periods of acute or chronic illness to mitigate muscle degradation, enhance recovery, and reduce adiposity-associated inflammation [26,28].

The quality, quantity, and distribution of protein intake are important for maximizing muscle protein synthesis (MPS) and maintaining bone health. High-quality proteins rich in essential amino acids (EAAs), particularly leucine, stimulate the mTORC1 pathway, a key regulator of muscle anabolism. Several data indicate that activation of this pathway could enhance osteoblast activity and inhibit osteoclast-mediated bone resorption, leading to downstream effects on bone density [29,30]. Current recommendations encourage the consumption of ~25 to 30 g of protein with at least 2.5–3 g of leucine per meal to overcome anabolic resistance in older adults, optimize MPS, and promote bone health [31,32,33,34].

Leucine-enriched supplements, including β-hydroxy-β-methylbutyrate (HMB), have shown promise in reducing muscle catabolism during periods of inactivity or stress, such as hospitalization [35,36]. HMB not only enhances protein turnover but also reduces inflammatory markers like TNF-α, which contribute to both muscle wasting and bone loss [37,38]. The benefits of HMB supplementation are most pronounced when combined with resistance training, which synergistically improves muscle mass, bone density, and metabolic health [38]. Further evidence suggests a dose–response relationship for HMB up to 3 g/day, especially when paired with structured PA [38].

Interestingly, both timing and distribution of protein intake are critical. Research shows that an even distribution of proteins across meals maximizes the anabolic response and supports bone remodeling [39,40]. Post-exercise protein ingestion enhances MPS and muscle recovery while promoting calcium absorption and bone repair [41,42]. Further emerging evidence highlights the role of co-supplementation with amino acids such as arginine, lysine, and glutamine in enhancing muscle quality and bone integrity. Arginine improves endothelial function and nitric oxide production, which are critical for muscle perfusion, recovery, and bone vascularization. Lysine and glutamine exhibit protective effects against muscle wasting and support collagen synthesis, further complementing OS management strategies [39,40]. In addition, anti-inflammatory nutrients such as omega-3 fatty acids can enhance musculoskeletal benefits when combined with protein. This dual action supports both muscle maintenance and bone density, providing comprehensive benefits in the management of OS [41,42,43].

Recent studies suggest that protein quality significantly impacts its anabolic potential. Fast-digesting proteins, such as whey, provide a rapid rise in amino acids, particularly leucine, in the bloodstream, enhancing MPS and osteoblast activity. Conversely, mixed meals containing slower-digesting proteins or lower-quality plant proteins may require higher doses or fortification with EAAs to achieve similar anabolic and osteogenic effects. Combining different protein sources improves the overall amino acid profile, optimizing their anabolic and bone-supporting properties [44,45,46]. To overcome the challenges of anabolic resistance and inflammation, innovative strategies such as leucine fortification of meals or supplementation with high-quality protein are critical. These approaches are particularly relevant for older adults experiencing appetite loss, dietary restrictions, or obesity-associated inflammation. Studies suggest that leucine-enriched diets can stimulate MPS and reduce inflammatory markers even in suboptimal protein conditions, offering practical solutions for managing OS in older populations [47].

### 5.2. Vitamins and Minerals

The nutritional management of OS requires an integrated approach that includes an optimal intake of essential vitamins and minerals, besides protein, to support bone and muscle tissues. Certain micronutrients play a pivotal role in modulating the bone–muscle axis, also addressing chronic inflammation, endocrine dysfunction, and metabolic dysregulation associated with OS.

### 5.3. Calcium and Vitamin D

Calcium is a cornerstone nutrient for bone health, necessary for maintaining bone mineral density and preventing osteoporosis. Vitamin D complements calcium by facilitating its absorption in the intestine and regulating bone remodeling through the calcium–parathyroid hormone axis. Deficiencies in vitamin D have been strongly correlated with reduced muscle strength and increased fat mass. Current guidelines recommend a daily intake of 1000–1200 mg of calcium and 800–1000 IU of vitamin D, with higher doses considered in cases of deficiency or increased requirements. Additionally, the sequestration of vitamin D by adipose tissue and its reduced bioavailability in older individuals with visceral obesity necessitate careful assessment and potential supplementation in OS populations [48,49,50,51]. It must also be mentioned that sun exposure is not sufficient to guarantee a sufficient serum level of Vitamin D above the recommended values. Thus, Vitamin D integration must always be considered in individuals affected by OS.

### 5.4. Minerals (Mg, I, K)

Magnesium is a cofactor in over 300 enzymatic reactions, including those related to bone and muscle health. It supports bone mineralization, modulates inflammatory markers, and is critical for muscle function and recovery. Suboptimal magnesium levels are associated with increased fat mass, muscle weakness, and low bone density. Recommended daily intake ranges from 310 to 420 mg, with dietary sources including nuts, seeds, whole grains, and leafy green vegetables [52,53].

Iron is essential for oxygen transport and energy production in muscle tissue, while zinc is a critical component of bone matrix proteins and supports muscle repair and regeneration. Both minerals are involved in reducing oxidative stress and inflammation, which are key drivers of OS. Optimal intake of these minerals from dietary sources includes lean meats, seafood, legumes, and fortified cereals [54,55].

Potassium contributes to the maintenance of acid–base balance, which is critical for bone mineralization, and supports muscle contraction and nerve function. Its anti-inflammatory properties also make it a valuable nutrient for managing OS. Potassium-rich foods include bananas, potatoes, citrus fruits, and beans [56,57].

### 5.5. Vitamin K

Vitamin K, particularly in its K_2_ form, is essential for activating osteocalcin, a protein that binds calcium in the bone matrix, thus improving bone density and reducing the risk of fractures. It also plays a role in muscle health by modulating mitochondrial function and energy metabolism. Foods such as fermented soy products, dairy, and green leafy vegetables are excellent sources of vitamin K_2_ [58,59].

### 5.6. B-Vitamins

The B-vitamin group, especially B_6_, B_9_ (folate), and B_12_, supports muscle and bone health by regulating homocysteine levels, which, when elevated, can negatively impact bone strength and vascularization of muscle tissue. Adequate intake of these vitamins is critical for reducing systemic inflammation and supporting protein metabolism [60,61].

### 5.7. Vitamins C and E

Vitamins C and E provide antioxidant protection against oxidative damage in bone and muscle tissues. Vitamin C is particularly important for collagen synthesis, a major component of the bone matrix, and supports immune function. Vitamin E enhances mitochondrial efficiency and reduces lipid peroxidation in muscle cells, improving recovery and strength [62,63].

### 5.8. Omega-3 Fatty Acids

Recent evidence suggests that omega-3 fatty acids (n3-PUFA) from fish oil can enhance the anabolic response of muscles to resistance training and improve physical performance in older adults with OS. Interestingly, n-3 PUFA supplementation might help older individuals affected by fragility as compared to healthy subjects [64]. Moreover, further research suggests that n-3 PUFA might exert their positive effects by enhancing the mTORC-1 signaling pathway to other stimuli, more than directly activate muscle protein synthesis [65].

These nutrients also exhibit anti-inflammatory properties (as further discussed below), making them valuable in managing chronic low-grade inflammation [66,67]. Interestingly, even though there are no specific studies demonstrating a role of PUFA on osteoporosis prevention, a recent NANES study demonstrated a significant inverse relationship between osteoporosis risk and dietary n3 PUFA intake, suggesting that they might play a crucial role in bone tissue homeostasis [68].

## 6. Clinical Considerations and Strategies for Modulating Inflammation in OS

**Figure 2:**
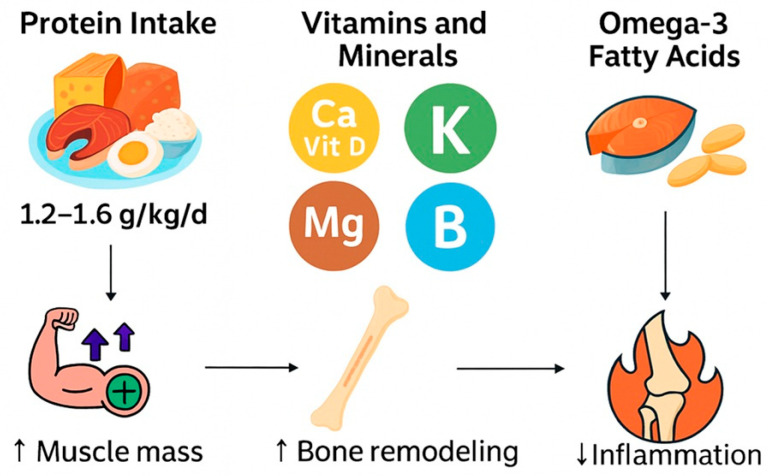
Nutritional strategies for the management of OS.

In individuals with OS, ensuring adequate intake of these vitamins and minerals through diet can be challenging due to dietary restrictions, low appetite, or malabsorption issues. Targeted supplementation may be required to achieve optimal levels, particularly for nutrients like vitamin D, calcium, and magnesium. Co-supplementation strategies, such as combining calcium and vitamin D or pairing antioxidants with anti-inflammatory nutrients (Figure 2), can further enhance therapeutic outcomes. Regular monitoring and tailoring interventions to individual needs are critical for maximizing benefits [69,70].

Inflammation plays a central role in the pathogenesis and progression of OS [70]. Chronic low-grade inflammation, commonly referred to as “inflammaging,” is a hallmark of aging and might operate as a pivotal mechanism exacerbating muscle degradation, bone resorption, and adipose tissue dysfunction [70]. This chronic subclinical inflammation is driven by the secretion of pro-inflammatory cytokines such as TNF-α, IL-6, and IL-1β, produced primarily by adipose tissue macrophages and other immune cells infiltrating dysfunctional adipose tissue [71,72].

Chronic inflammation contributes to anabolic resistance and promotes muscle protein breakdown via the ubiquitin–proteasome pathway. Elevated levels of TNF-α and IL-6 impair satellite cell function and mitochondrial activity, leading to sarcopenia and reduced muscle strength [73]. Further, pro-inflammatory cytokines stimulate osteoclastogenesis while suppressing osteoblast activity, resulting in accelerated bone resorption and reduced bone formation. Dysregulation in pathways such as the RANK/RANKL/osteoprotegerin axis further exacerbates bone fragility [74]. This imbalance amplifies systemic inflammation and metabolic dysregulation, worsening the interplay between muscle and bone health [75].

### 6.1. Antioxidant-Rich Diets and Lifestyle Modification

A diet rich in anti-inflammatory foods, such as omega-3 fatty acids, polyphenols, and fiber, can help reduce systemic inflammation [76].

The Omega-3 Fatty Acids, polyunsaturated fats, exhibit anti-inflammatory properties by reducing the production of pro-inflammatory eicosanoids and cytokines. Supplementation has been shown to improve muscle protein synthesis and bone density while mitigating systemic inflammation [77,78]. Interestingly, proteins exhibit protective anti-inflammaging effects against muscle wasting and support collagen synthesis, further complementing OS management strategies [39,40]. In particular, studies suggest that specific amino acid integration, such as leucine, can stimulate MPS and decrease levels of inflammatory markers even in suboptimal dietary protein conditions, offering real-world solutions to manage OS in aged populations.

Beyond its role in calcium metabolism, vitamin D modulates immune responses by decreasing TNF-α and IL-6 levels and promoting regulatory T-cell activity. Ensuring sufficient vitamin D levels may help mitigate inflammatory processes in OS, which, as previously mentioned, might also contribute to worsening the pathological mechanism(s) involved in sarcopenia development [78]. A diet rich in fruits, vegetables, and whole grains provides vitamins C and E, polyphenols, and flavonoids, which neutralize oxidative stress and reduce inflammatory markers [79].

Furthermore, it is interesting to remind that adequate sleep and stress management are critical, as disrupted circadian rhythms and chronic stress can exacerbate inflammatory processes through dysregulation of the hypothalamic–pituitary–adrenal axis [80,81]. Also, regular physical activity exerts potent anti-inflammatory effects by reducing circulating cytokines, improving mitochondrial function, and enhancing insulin sensitivity, making it a cornerstone intervention in the management of OS-related inflammation [82].

### 6.2. Energy and Nutrient Intake in the Treatment of OS

Energy requirements in OS are influenced by the loss of lean mass, adaptive thermogenesis, and metabolic adaptations associated with increased adipose tissue and sarcopenia. A precise caloric adjustment is essential to avoid exacerbating sarcopenia or promoting excessive fat accumulation, leading to obesity [83]. However, severe caloric restrictions and fasting protocols are particularly dangerous in this population, as they can accelerate muscle catabolism, compromise bone health, and lead to metabolic dysregulation. If necessary, a carefully calibrated caloric deficit, typically 500 kcal/day, supports fat loss while preserving lean mass and metabolic stability [84].

Protein metabolism plays a central role in the management of OS. Protein intake is critical, with recommendations of 1.2–1.5 g/kg of ideal body weight per day to stimulate MPS and counteract sarcopenia [85]. High-quality protein sources rich in essential amino acids, particularly leucine, such as dairy, eggs, and lean meats, should be emphasized to promote anabolism [86]. Frequent, evenly distributed protein intake across meals (e.g., ~20 to 30 g per meal) further optimizes MPS and prevents prolonged periods of muscle protein breakdown [87].

Caloric balance strategies must also focus on macronutrient composition to support metabolic and functional outcomes. Complex carbohydrates, constituting about 45–55% of total energy intake, provide sustained energy for PA and enhance insulin sensitivity, a critical factor in preserving muscle mass [88,89]. Dietary fats, contributing roughly 25–30% of total energy intake, should prioritize unsaturated fatty acids to modulate inflammation and support cardiovascular health. Micronutrient adequacy remains vital: calcium (1000–1200 mg/day) and vitamin D (800–1000 IU/day) are particularly important to mitigate bone loss, while magnesium, vitamin K, and omega-3 fatty acids contribute to overall bone and muscle health [88].

To promote an anabolic state, dietary strategies should be paired with resistance and weight-bearing exercises [88]. Such activities stimulate muscle hypertrophy and bone formation, counteracting the catabolic effects of obesity and sarcopenia. Energy intake must align with increased energy demands from exercise to support recovery and adaptation. The inclusion of post-exercise protein supplementation may further enhance muscle repair and growth [89].

Regular monitoring of body composition, including lean mass, fat mass, and bone mineral density, is critical to guide caloric and macronutrient adjustments [90]. Dynamic energy needs must be addressed to avoid unintentional caloric deficits or surpluses that could undermine treatment goals. A gradual, sustainable approach to caloric balance not only optimizes clinical outcomes but also reduces the risk of adverse effects associated with drastic dietary interventions.

## 7. Physical Activity

Physical exercise is widely recommended as both a preventive and non-pharmacological therapeutic intervention for OS [91].

**Figure 3:**
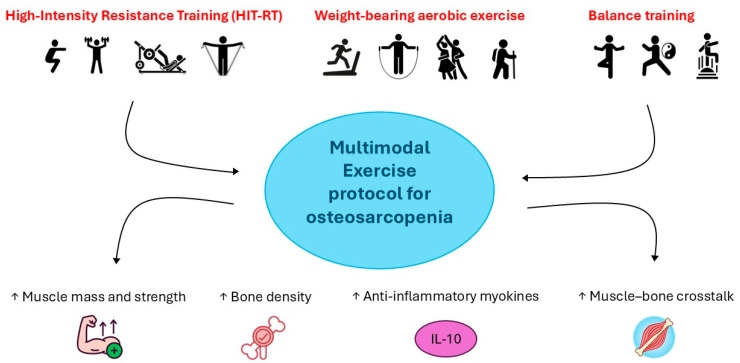
Multimodal Exercise Protocol for the Management of OS.

Structured exercise programs tailored to individual capacities play a pivotal role in managing OS by improving muscle mass, bone density, and overall physical performance (Figure 3) [92]. Exercise also reduces adipose tissue inflammation, enhances the release of anti-inflammatory myokines, such as IL-10, and improves muscle–bone crosstalk [92]. Several relevant cytokines, “exerkines” [93] such as irisin, are produced and released from skeletal muscle for mediating systemic adaptations, as an exercise-dependent response. Interestingly, preclinical studies indicate that exercise mitigates sarcopenia by modulating multiple intracellular signaling pathways. Indeed, resistance training appears to prevent muscle fibrosis and atrophy by down-regulating C1q-induced Wnt signaling or PI3K-Akt-TSC signaling cascade leading to mTORC1 signaling regulation, while aerobic exercise activates the AMPK/PGC-1α signaling pathway promoting mitochondrial quality control. Further, the increase in circulating levels of irisin appears to enhance the expression of UCP1, thereby stimulating the conversion of white fat into brown fat and improving metabolic pattern [94].

Thus, on the basis of the preclinical research data, a multimodal exercise protocol that integrates resistance training, weight-bearing aerobic exercise, and balance training is preferable to single-modality programs [92]. Such an integrated approach targets the various pathophysiological aspects of OS, supporting musculoskeletal strength, cardiovascular health, postural control, and neuromuscular coordination (Figure 3).

Resistance training is a cornerstone in the management of OS, due to its ability to counteract muscle atrophy and improve strength [95,96]. This exercise type involves muscle contractions against an external resistance, such as free weights, resistance bands, or body weight. Progressive resistance training was strongly recommended by the International Conference on Sarcopenia and Frailty Research (ICSFR) as a first-line therapy for obtaining muscle hypertrophy, strength gain, and improved physical performance [97]. Evidence from the Franconian Osteopenia and Sarcopenia Trial (FrOST) highlights the efficacy of high-intensity resistance training (HIT-RT) combined with nutritional supplementation. This intervention included supervised, machine-based dynamic resistance exercises performed twice weekly, targeting all major and minor muscle groups. Intensity was guided by repetition ranges (5–7 or 8–10) and work to non-repetition maximum (nRM), resulting in the preservation of lumbar spine bone mineral density (LS-BMD) and a significant increase in skeletal muscle index (SMI), a muscle mass parameter, over a 12-month period in elderly men with OS [96]. Furthermore, even low-load resistance training, performed at intensities up to 50% of one-repetition maximum (1RM), has demonstrated substantial improvements in muscle strength and functional outcomes among older adults [92,98], making it a viable option for individuals with limited physical capacity.

Weight-bearing aerobic exercise is a key complementary intervention to resistance training, as it promotes the maintenance of both muscle and cardiorespiratory function, thereby contributing to bone integrity. These exercises involve working against gravity and include activities such as walking, cycling, stair climbing, jumping, jogging, dancing, and tai chi. Recommended training programs typically consist of at least 50 weight-bearing impacts per session, ranging from high (i.e., jogging) and moderate (i.e., stair climbing) to low (i.e., walking) impact, performed on most days of the week, with the goal of preserving musculoskeletal health [99]. High-impact exercises such as jumping, jogging, and step aerobics generate greater ground reaction forces and have been shown to significantly improve bone mineral density (BMD) [100]. In contrast, low-impact exercises such as brisk walking, low-impact dance, and tai chi produce more moderate osteogenic stimuli and offer a safer alternative for older adults or those at higher risk of fracture [99].

Balance training is particularly important to prevent falls, which are a major cause of morbidity and mortality in individuals with OS [101]. Exercises that challenge proprioception and postural stability, such as tandem walking, single-leg stands, and tai chi, not only enhance balance but also contribute to neuromuscular coordination, thereby reducing fall risk [92,101]. Moreover, balance training engages multiple muscle groups, particularly promoting endurance of antigravity muscles [92].

In summary, a combined exercise protocol that includes resistance training, weight-bearing aerobic activity, and balance training is essential for the effective management of OS. These exercise modalities, when combined, offer complementary benefits that enhance musculoskeletal strength, maintain cardiovascular health, and reduce the risk of falls.

## 8. Exercise and Nutritional Interactions

In the management of OS, integrating nutritional therapy with physical exercise is essential, as diet and activity synergistically affect adipose, muscle, and bone tissues. Resistance training is a cornerstone for counteracting muscle atrophy and bone loss, but its benefits are amplified only when supported by adequate caloric and protein intake. Clinical studies in older adults with OS show that resistance training alone increases lean mass (~3% on average) and muscle strength, whereas combining exercise with higher protein intake leads to greater improvements in body composition (notably, more pronounced fat mass reduction) than exercise alone [102]. Accordingly, guidelines recommend protein intakes above the RDA for active older individuals, approximately 1.0–1.2 g/kg/day for healthy elderly, and up to 1.5 g/kg/day in cases of intense exercise or caloric restriction to prevent muscle loss during weight reduction. Practically, ingesting high-leucine protein immediately post-exercise supports muscle protein resynthesis and enhances the neuromuscular adaptations triggered by training. In a randomized trial of sarcopenic women over 75 years old, those performing resistance training combined with essential amino acid supplementation (3 g leucine twice daily) showed significantly greater gains in lower-limb muscle mass and strength compared to exercise alone [103]. Beyond protein, other nutrients also interact beneficially with exercise. For example, omega-3 fatty acid supplementation (EPA/DHA) in older adults increased MPS rates and may enhance anabolic responsiveness to resistance training [104]. To maximize skeletal health, weight-bearing and impact-loading exercises should be accompanied by sufficient calcium and vitamin D intake, which are crucial for bone health in general and particularly in OS. Overall, an integrated approach combining structured physical activity (resistance, aerobic, balance training) with a targeted, protein-rich diet, properly timed and micronutrient-replete, is considered the most effective strategy to improve body composition and physical function in patients with OS.

## 9. Challenges and Strategies in the Implementation of Lifestyle Interventions for OS

Implementing structured nutrition and exercise programs in patients with osteosarcopenia can be challenging, as many individuals present with multiple comorbidities (e.g., cardiovascular disease, diabetes, arthritis) and chronic musculoskeletal pain that limits mobility, reduces exercise tolerance, and increases fatigue. Psychological barriers, such as fear of pain or injury, and logistical constraints (i.e., lack of access to supervised programs), further complicate adherence.

When standard exercise protocols are not feasible, alternative low-impact exercise approaches can help preserve muscle and bone function.

Neuromuscular electrical stimulation (NMES) involves the application of surface electrodes to deliver electrical impulses that elicit involuntary muscle contractions, mimicking some of the physiological effects of active exercise. According to O’Connor et al. (2018), NMES represents a valuable adjunct or alternative to conventional training in older adults with sarcopenia, and by extension OS, particularly for those unable to engage in traditional exercise due to pain, comorbidities, or severe mobility restrictions [105].

Blood flow restriction (BFR) training is a technique in which venous return from the targeted muscles is partially occluded while arterial inflow is preserved, typically through the application of pneumatic cuffs or elastic bands. This controlled restriction creates a localized hypoxic environment and promotes the accumulation of metabolites such as lactate, which in turn stimulates growth hormone release, fast-twitch fiber recruitment, and the activation of hypertrophic signaling pathways (notably mTORC1 and MAPK). These mechanisms enable significant gains in muscle strength and hypertrophy even when training at low intensities (≥20% 1RM), making BFR a safer alternative for individuals unable to tolerate high-load resistance training. Importantly, in populations with joint pain, frailty, or OS, BFR offers a way to achieve meaningful musculoskeletal adaptations while minimizing joint stress and reducing injury risk [106].

Whole-body vibration therapy (WBV) provides mechanical stimuli that can promote bone remodeling and muscle activation at a lower perceived effort. For frail patients who struggle with conventional physical exercise, vibration therapy has emerged as a promising alternative [107].

Collectively, these modalities offer a multimodal, progressive approach to maintain musculoskeletal health in OS patients with functional limitations, supporting mitochondrial activity, reducing fatigue, alleviating pain, and facilitating the gradual reintroduction of more traditional forms of exercise.

Last, it is worth mentioning the potential role that a healthy microbiota might exert in preventing or treating OS [108,109]. Indeed, it is known that during the aging process, an alteration in the microbiota population occurs with an increased presence of dysbiosis. Thus, even though few studies have addressed this topic, potential new strategies of intervention might be aimed at the modulation of both gut and oral microbiota by adopting healthy dietary patterns with an adequate protein intake, supplementation with prebiotics, probiotics, and n3PUFA combined with physical exercise to prevent or correct OS.

## 10. Conclusions

OS is a complex clinical challenge arising from the detrimental interplay among muscle loss, bone fragility, and, also, increased adiposity. Recognizing OS as an integrated syndrome requires a paradigm shift in diagnosis and management, moving beyond compartmentalized approaches toward multimodal strategies that simultaneously address the pathogenic mechanisms as well as clinical consequences of musculoskeletal fragility.

Current scientific evidence strongly supports the implementation of a structured nutritional intervention, including adequate protein intake evenly distributed across meals, targeted supplementation with high-quality amino acid sources, and careful regulation of energy and micronutrient intake. This must be complemented by a personalized PA program emphasizing resistance and weight-bearing exercises, capable of simultaneously promoting muscle hypertrophy, bone remodeling, and metabolic improvement.

Moreover, managing chronic low-grade inflammation and associated metabolic comorbidities through dietary, behavioral, and, where appropriate, pharmacological interventions is essential. In this context, an interdisciplinary approach integrating physicians, dietitians, physiotherapists, and other healthcare professionals creates the foundation for improving quality of life and functional independence in aged individuals affected by OS.

## 11. Strengths and Limitations

This review has several strengths. First, it addresses OS from an integrated perspective, discussing both nutritional and PA interventions within the same conceptual framework. This approach reflects the current understanding of OS as a unified syndrome, emphasizing the interaction between muscle, bone, and adipose tissue. Second, the synthesis includes evidence from both randomized controlled trials and observational studies, thus offering a comprehensive overview of available strategies. In addition, the inclusion of graphical summaries facilitates the accessibility of complex information for both clinicians and researchers.

However, some limitations need to be recognized. The number of studies specifically targeting OS remains limited, and much of the current evidence is extrapolated only from osteoporosis or sarcopenia studies. The heterogeneity of diagnostic criteria, intervention protocols, and outcome measures across studies limits the ability to directly compare results and draw definitive conclusions.

## 12. Practical Guidance for Clinical Application

To bridge the gap between evidence and implementation, it is essential to provide clear, actionable recommendations that can be easily applied in different healthcare settings. Thus, practical examples of exercise protocols and nutritional strategies for individuals with OS, adapted from established guidelines for sarcopenia and osteoporosis, are summarized in Table 1. These tools are intended to support clinicians in designing comprehensive, individualized interventions aimed at improving muscle mass, bone density, functional performance, and overall quality of life in affected patients.

## Figures and Tables

**Table 1:** Practical recommendations for exercise prescription for people with OS.

Type	Frequency	Modality	Intensity	Volume
Resistance Training	2 days/w	Machine-based or free-weight exercises for major and minor muscle groups (e.g., leg press, chest press, seated row)	From moderate (50% 1RM) to high intensity (80% 1RM) (progressive overload)	2–3 sets of 8–12 reps
Weight-Bearing Aerobic Activity	Most days	Brisk walking, stair climbing, dance, jogging, jumping	Low-moderate (for those at higher risk of fracture) to high impact (for those at lower fracture risk)	50 weight-bearing impacts per session (5 sets of 10 with reduced impacts in between). 20 min session if only lower impact advised
Balance and Coordination	2–3 days/w For fallers—most days	Tai chi, tandem walking, single-leg stands	Moderate to highly challenging gradual progression	3 h a week (25 min/day or 3 × 1 h sessions a week)
